# De-prescribing strategy in a case of Delirium in the elderly

**DOI:** 10.1192/j.eurpsy.2022.1671

**Published:** 2022-09-01

**Authors:** B. Díez Valle, P. Coucheiro Limeres, J. Roldán Larreta

**Affiliations:** 1 Hospital Universitario Severo Ochoa, Psychiatry, Leganés, Spain; 2 Hospital Universitario José Germain, Psychiatry Department, Leganés, Spain; 3 Clínica Josefina Arregui, Psychogeriatrics, Alsasua, Spain

**Keywords:** delirium, De-prescribing, prescription cascade, Polypharmacy

## Abstract

**Introduction:**

Iatrogenic factors, such as polypharmacy and prescription cascade, are some of the main causes of delirium in the elderly. We present a case of delirium of months of evolution that improved after applying a pharmacological de-prescription strategy.

**Objectives:**

To report a case and review the available literature on the concepts of prescription cascade and de-prescription in delirium in the elderly.

**Methods:**

A 92-year-old woman with a history of cerebrovascular accidents and no psychiatric history or dementia was admitted to a psychogeriatric clinic due to disorientation, delusions of harm and gait apraxia. Several months earlier she had required admission to the general hospital for agitation. In view of the suspicion of delirium, an exhaustive examination and complementary tests were performed, including a neuropsychological assessment and a brain scan (Image 1).

**Figure:**
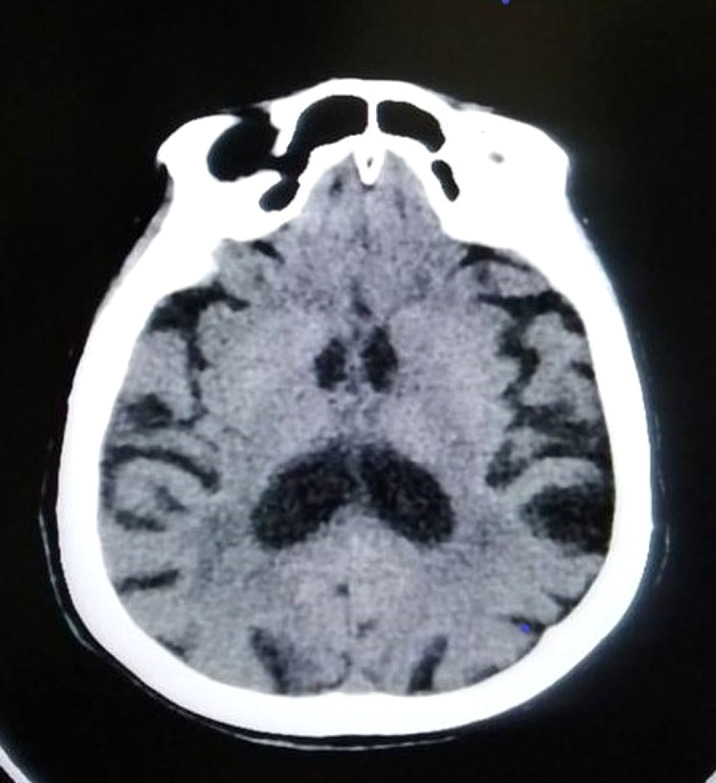

**Results:**

The patient had previously received multiple high-dose psychotropic drugs (Gabapentin, trazodone, Zolpidem, Quetiapine), which had reduced the agitation but had not resolved the problem. Organic causes were treated in a multidisciplinary team (pressure ulcers), together with a gradual tapering of medication. Although underlying vascular dementia was diagnosed, the patient’s gait and cognitive status improved, with a significant impact on her autonomy and quality of life.

**Conclusions:**

Despite an extensive literature on the subject, delirium in the elderly remains an under-diagnosed medical condition, especially the hypoactive subtype, just as cascade prescribing remains common. It is important to raise awareness among specialists in training to prevent and diagnose it.

**Disclosure:**

No significant relationships.

